# Plasma Matrix Metalloproteinase-9 and Tissue Inhibitor of Matrix Metalloproteinase-1 as Prognostic Biomarkers in Critically Ill Patients

**DOI:** 10.1515/med-2020-0008

**Published:** 2020-03-04

**Authors:** Izabela Duda, Łukasz Krzych, Halina Jędrzejowska-Szypułka, Joana Lewin-Kowalik

**Affiliations:** 1 Medical University of Silesia School of Medicine in Katowice, Katowice, Poland; 2 Department of Anesthesiology and Intensive Care, Faculty of Medicine in Katowice, Medical University of Silesia in Katowice Katowice, Poland; 3 Department of Physiology, Faculty of Medicine in Katowice, Medical University of Silesia in Katowice, Katowice Poland

**Keywords:** Matrix metalloproteinase 9, Tissue inhibitor of metalloproteinase 1, Biomarker, multiple organ failure

## Abstract

Matrix metalloproteinase 9 (MMP-9) plays an important role in inflammatory and pathological processes by enabling the inflow of leukocytes to the site of infection or tissue damage. MMP-9 and tissue inhibitor of metalloproteinase 1 (TIMP-1) have been described as potential prognostic biomarkers in various clinical settings. The aim of the study was to evaluate the usefulness of plasma levels of MMP-9 and TIMP-1 as well as the MMP-9/ TIMP-1 ratio in predicting the outcome in patients admitted to the intensive care unit (ICU). The study included 56 critically ill patients with multiple organ failure. Plasma levels of MMP-9 and TIMP-1 were determined on hospitalization day 1, 2, 3 and 7. Nineteen (35.7%) patients died. The level of TIMP-1 was statistically significantly higher on day 1 and 7 of hospitalization in non-survivors, as compared to survivors (p=0.01). A statistically significant positive correlation was found between MMP-9 and TIMP-1. The MMP-9/TIMP-1 ratio was comparable in both groups during of observation (0.62 on day 1). The MMP-9/TIMP-1 ratio was positively correlated with the level of lactate and negatively correlated with platelet count. Likewise, TIMP-1 was positively correlated with the level of lactate. The level of MMP-9 was higher in the non-survivor group only on day 7 of observation. In conclusion, although TIMP-1 and MMP-9 concentrations were higher in non-survivors and the MMP-9/TIMP-1 ratio was related to some parameters of critical illness, further research is needed to verify whether they can serve as reliable biomarkers for early prognostication of ICU patients.

## 1 Introduction

The extracellular matrix (ECM) is a dynamic, elastic and compound structure that fills the area between cells [1]. Matrix metalloproteinases (MMPs) belong to the group of enzymes involved in the degradation of basilar membrane proteins and ECM, which in turn facilitates the migration of cells. Metalloproteinases are produced by the majority of connective tissue cells, leukocytes, macrophages, vascular endothelial cells and neoplastic cells. After their release into the extracellular matrix, MMPs remain inactive. Their activation occurs through the cleavage of cysteine mediated by some proteolytic enzymes (plasmin, thrombin) and already active MMPs. The activity of MMPs is inhibited by specific tissue inhibitors of metalloproteinase (TIMP-1 - TIMP-4) and non-specific plasma inhibitors.

MMPs belong to the family of multidomain proteolytic enzymes containing zinc ions. They are divided into the following subgroups: matrilizines, collagenases, stromelysines, gelatinases, membrane-type matrix metalloproteinases and other matrix metalloproteinases [2, 3, 4]. MMP-9 (gelatinase B) belongs to a group of collagenases and plays an important role in inflammatory and pathological reactions, enabling the inflow of leukocytes to the site of infection or tissue damage through degradation of basilar membrane components and ECM as well as activation of cytokines and chemokines [5, 6]. Its specific inhibitor is TIMP-1. The MMP-9/TIMP-1 expression ratio defines the activity of MMP-9.

Both MMP-9 and TIMP-1 can be used as potential biomarkers of the severity of inflammation and tissue damage and for prognostication. MMP-9 has been found in such pathological processes as neoplasms, immunological and cardiovascular diseases as well as malaria in pregnant women [7,8,9]. There are several reports demonstrating an increase in MMP-9 and TIMP-1 levels in sepsis [10,11,12,13,14]. However, the usefulness of MMP-9 and TIMP-1 in predicting the mortality of ICU patients has not been fully elucidated.

The study hypothesis was that the levels of MMP-9 and TIMP-1 were elevated in patients hospitalised in the ICU due to multiple organ failure (MOF) and that the severity of organ dysfunction and treatment outcomes were correlated with the levels of MMP-9 and TIMP-1. Therefore, our aim was to evaluate the usefulness of MMP-9/TIMP-1 ratio as an early biomarker for risk assessment in critically ill patients.

## 2 Material and methods

### 2.1 Patients

The study was performed in the 10-bed mixed-profile intensive care unit (ICU) for adults. The study design was approved by the Bioethics Committee of the Medical University of Silesia in Katowice (KNW/0022/KB/208/15); patient consent for participation in the study was not required. The study covered the period of 6 months-between October 2015 and March 2016.

**Figure 1:**
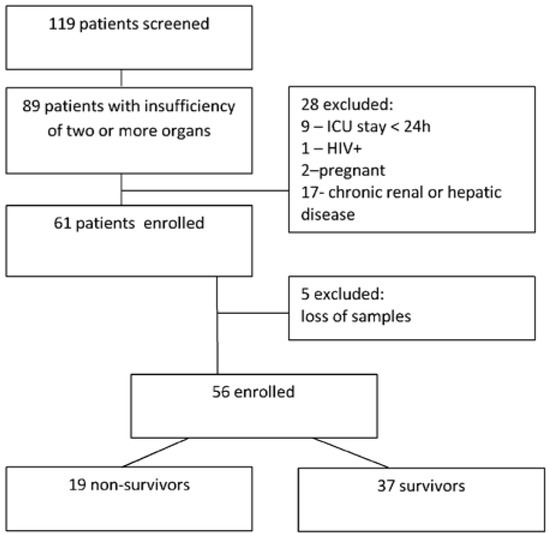
Flow chart of patients included in the study.

All the patients admitted to the ICU were evaluated in terms of the symptoms of multiple organ failure. The study included patients with the failure of at least two organs. The failure of the circulatory system was diagnosed when vasopressors or positive inotropic drugs were required; the respiratory failure was defined as PaO_2_/FiO_2_ < 250; the nervous failure was diagnosed at GCS < 10 points, kidney failure at diuresis < 0.5 ml/kg/h for > 6h, haematological failure at platelet count < 80 000/mm^3^ or metabolic acidosis with pH < 7.3 and lactate concentration > 1.5 x exceeding the upper normal limit. The exclusion criteria were age <18 years, pregnant patients, high mortality risk within the next 24 hours, chronic liver or kidney failure, human immunodeficiency virus (HIV) or other causes of immunosuppression (Fig.1). The inclusion to the study had no influence on the therapeutic mode. All the patients were treated according to the typical ICU management protocol. The information collected from patients included demographic data, concomitant diseases, duration of ICU stay, type of admission to ICU (medical or surgical). The severity of diseases on admission was determined according to the Acute Physiology and Chronic Health Evaluation II (APACHE II). The examinations were discontinued when the patient died or was discharged from ICU. Mortality was defined as a patient’s death within 28 days after admission to ICU.

### 2.2 Laboratory analysis

All laboratory tests were performed immediately after ICU admission. The following parameters were recorded: levels of creatinine and bilirubin, white blood cell count, levels of lactate, platelet count, levels of haemoglobin and C-reactive protein.

The venous blood for MMP-9 and TIMP-1 determinations was sampled on day 1, 2, 3 and 7. The collected sample was immediately centrifuged at 3000g for 15 minutes and the supernatant was frozen at -80^o^C until analysis. MMP-9 and TIMP-1 were determined using a commercial ELISA kit (Cloud-Clone Corp. USA) according to the manufacturer`s instructions.

### 2.3 Statistical analysis

Statistical analysis was performed using MedCalc Statistical Software version 17.7 (MedCalc Software bvba, Ostend, Belgium; http://www.medcalc.org; 2017). Quantitative variables were presented as the median and interquartile range (IQR; 25pc-75pc) while qualitative variables as the absolute value and percentage. The distribution of quantitative variables was verified by the Shapiro-Wilk test. The intergroup differences for quantitative variables were assessed using the Mann-Whitney U test (or the Kruskall-Wallis test). The Friedman test was applied for dependent variables. The intergroup differences for qualitative variables were evaluated based on the Spearman`s rank correlation coefficient. Diagnostic accuracy of MMP-9, TIMP-9 and MMP-9/TIMP-9 for prediction of mortality during ICU hospitalisation was assessed using receiver operating characteristic (ROC) curves. The threshold of statistical significance was assumed at α=0.05.

### 2.4 Results

**Figure 2:**
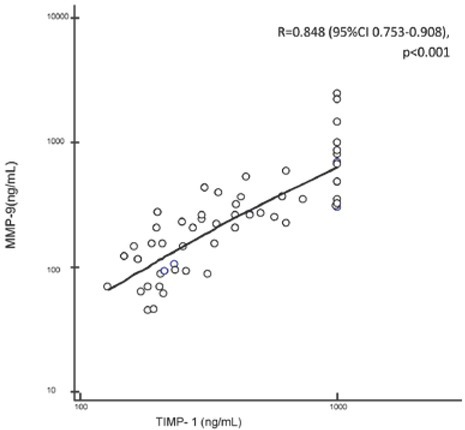
Correlation between MMP-9 and TIMP-1 on day 1 of ICU hospitalization. (MMP, matrix metalloproteinase; TIMP, tissue inhibitor of matrix metalloproteinase)

**Figure 3:**
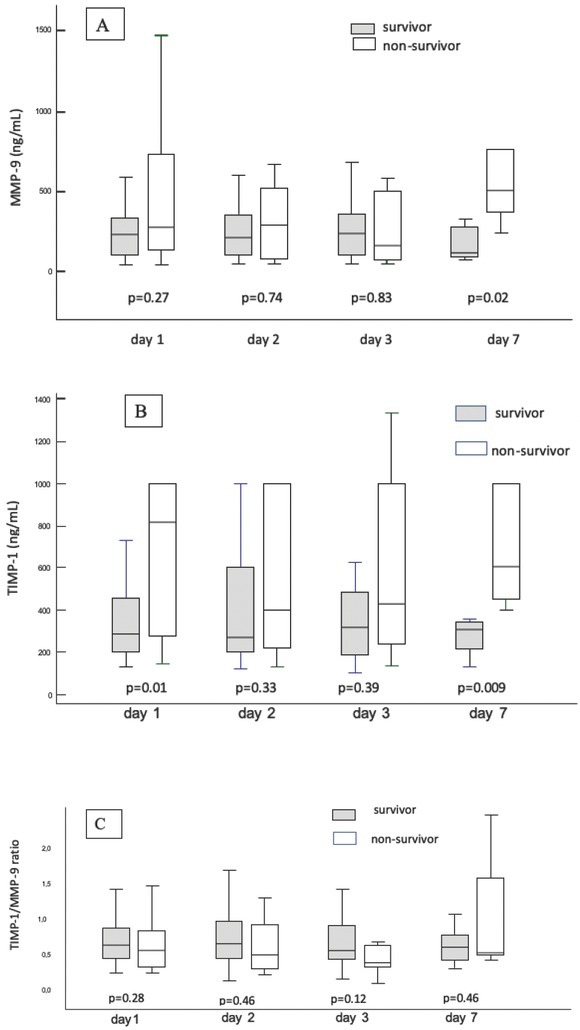
Serum MMP-9 (A) , TIMP-1 (B) and TIMP-1/MMP-9 (C) ratio in survivor and non-survivor patient (median; 25-75 percentiles). (MMP, matrix metalloproteinase; TIMP, tissue inhibitor of matrix metalloproteinase)

**Figure 4:**
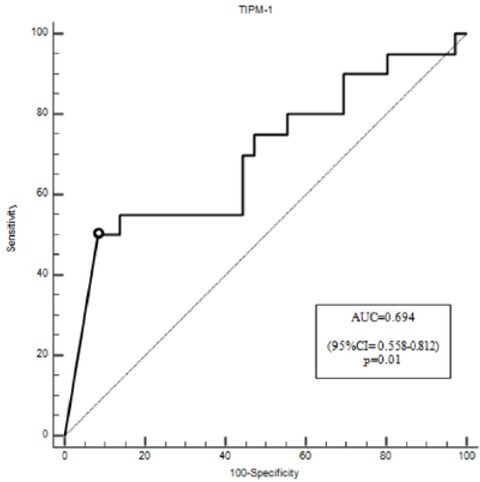
The receiver operating characteristic (ROC) curve for TIMP-1 on ICU day I as a predictor of mortality in critically ill patients. (TIMP, tissue inhibitor of matrix metalloproteinase).

Out of 119 screened patients, 56 patients with multiple organ failure were enrolled in the study (Figure 1). No differences in clinical and biochemical parameters were noticed between the group of non-survivors and survivors, except for platelet count, which was significantly lower in non-survivors (p=0.03). (Table 1) A strong statistically significant positive correlation was found between MMP-9 and TIMP-1 on days 1,2,3 and 7 (p<0.001) (Figure 2). Non-surviving patients showed higher serum TIMP-1 level on day 1 and higher serum levels TIMP-1 and MMP-9 on day 7 (Figure 3). The level of TIMP-1 on day 1 was found to be positively correlated with the levels of lactate and creatinine and negatively correlated with the concentration of hemoglobin. (Table 2). The MMP-9/TIMP-1 ratio was positively correlated with the level of lactate on day 2 of ICU stay and negatively correlated with platelet count on day 2. Additionally, the MMP-9/TIMP-1 ratio on day 1 was found to be negatively correlated with the duration of ICU stay (Table 3). 19 of 56 patients died resulting in a mortality rate of 35.7%. On ROC curve analysis, the area under the curve of TIMP-1 at day 1 to predict mortality were 0.694 (95% CI = 0.558-0.812; p=0.01) (Figure 4).

**Table 1:** Baseline clinical and biochemical characteristics of ICU survivors and non-survivors patients on day 1 of admission.

	All n=56	Survivors n=37	Non-survivors n=19	p
Age, years	61	61	65	0.94
	(48-70)	(49-69)	(48-71)	
Male sex	29	18	11	0.96
ICU days	8	8	8	0.94
	(4-14)	(4-14)	(4-16)	
APACHE II score	18	18	18	0.74
	(12-23)	(13-23)	(11-21)	
Lactate, mmol/L	2.85	2.4	3.3	0.16
	(1.9-5.1)	(1.9-3.5)	(2.35-6)	
Bilirubin, mg/dL	0.81	0,73	1.05	0.31
	(0.53-1.3)	(0.55-1.21)	(0.47-1.63)	
Creatinine, mg/dL	1.32	1.26	1.43	0.71
	(0.97-1.87)	(1.18-2.2)	(1.18-2.25)	
CRP, mg/L	172	153	201	0.94
	(99-245)	(100-243)	(93-286)	
Hemoglobin, g/dL	9.9	9.8	9.9	0.53
	(7.8-11)	(7.8-10.9)	(8.1-11.6)	
White blood cells,	15	13.3	17.3	0.15
10^9^ /L	(11-21)	(10.1-17.9)	(12.7-22)	
Platelets , 10^9^ /L	182	186	130	0.03
	(104-216)	(150-217)	(93-206)	
Causes of ICU admission, n; %				
Postoperative multiple organ failure	38			
	67.9%			
Acute neurological condition	7			
	12.5%			
Acute respiratory failure	5			
	8.9%			
Acute circulatory failure	4			
	7.1%			
Acute liver failure	1			
	1.8%			
Multiple trauma	1			
	1.8%			
MMP-9, ng/mL	241.3	235.1	281.5	0.3
	(192.4-295.7)	(110.8-337.3)	(138.5-736.6)	
TIMP-1, ng/mL	335.7	287.7	816.6	0.01
	(262.2-455)	(202.3-458.2)	(227.2-1000)	
MMP-9/TIMP-1, ratio	0.62	0.63	0.57	0.3
	(0.49-0.78)	(0.44-0.87)	(0.32-0.84)	

data are presented as median and interquartile range (in bracket) APACHE II , Acute Physiology and Chronic Health Evaluation; CRP , C-reactive protein; ICU, Intensive Care Unit; MMP-9, matrix metalloproteinase 9; TIMP-1, tissue inhibitor of matrix metalloproteinase 1

**Table 2:** Correlations between circulating MMP-9 and TIMP-1 with biochemical parameters on day 1.

	MMP-9	TIMP-1
MMP-9	NA	rho= 0.84; p<0.001
TIMP-1	rho= 0.84; p<0.001	NA
Bilirubin, mg/dL	rho= 0.16; p=0.23	rho= 0.28; p=0.1
CRP, mg/L	rho= 0.11; p=0.4	rho= 0.25; p=0.05
ICU days	rho= -0.23; p=0.08	rho= -0.1; p=0.46
Hemoglobin, g/dL	rho= -0.14; p=0.27	rho= -0.3 ; p=0.02
Creatinine, mg/dL	rho= 0.25; p=0.05	rho= 0.35; p=0.006
Lactate, mmol/L	rho= 0.14; p=0.27	rho= 0.35; p=0.007
Platelet, 109/L	rho= -0.02; p=0.85	rho= -0.22; p=0.1
WBC, 109/L	rho= 0.23; p=0.08	rho= 0.25; p=0.06
Age, years	rho= -0.1; p=0.42	rho= -0.048; p=0.72

CRP, C-reactive protein; ICU, Intensive Care Unit; MMP-9, matrix metalloproteinase 9; NA, not applicable; rho, rank-order correlation coefficient; TIMP-1, tissue inhibitor of matrix metalloproteinase 1; WBC, White Blood Cells

**Table 3:** Correlation between MMP-9/TIMP-1 ratio levels and bilirubin, CRP, hemoglobin, creatinine, lactate, platelet and ICU length of stay on days 1, 2 and 3.

	Day 1	Day 2	Day 3
Bilirubin, mg/dL	rho= 0.08: p=0.55	rho= 0.09; p=0.55	rho= -0.14; p=0.46
CRP, mg/L	rho= -0.14; p=0.29	rho= -0.21; p=0.18	rho= 0.21; p=0.28
ICU days	rho= -0.34; p=0.009	rho= -0.07; p=0.63	rho= 0.25; p=0.19
Hemoglobin, g/dL	rho= 0.14; p=0.27	rho= 0.3; p=0.14	rho= -0.2; p=0.18
Creatinine, mg/dL	rho= -0.004; p=0.97	rho= -0.24; p=0.13	rho= -0.18 p=0.35
Lactate, mmol/L	rho= -0.15; p=0.24	rho= -0.37; p=0.02	rho= -0.17; p=0.39
Platelet, 109/L	rho= 0.25; p=0.06	rho= 0.33; p=0.04	rho= 0.44; p=0.02
WBC, 109/L	rho= 0.01; p=0.94	rho= -0.106; p=0.52	rho= -0.007; p=0.97
Age, years	rho= -0.25; p=0.05	rho= -0.21; p=0.19	rho= -0.06; p=0.73

CRP, C-reactive protein; ICU, Intensive Care Unit; MMP-9, matrix metalloproteinase 9; rho, rank-order correlation coefficient; TIMP-1, tissue inhibitor of matrix metalloproteinase 1; WBC, White Blood Cells

## 3 Discussion

Our findings demonstrated that TIMP-1 and the MMP-9/ TIMP-1 ratio could be good diagnostic biomarkers in critically ill patients admitted to ICUs. This observation does not apply to MMP-9 which values did not correlate with laboratory inflammatory parameters of multiple organ failure and ICU treatment outcomes. TIMP -1 was significantly higher in non-survivors, as compared to survivors. However, the levels of MMP-9 and the MMP-9/TIMP-1 ratio were comparable in both groups. Several earlier studies have reported high levels of TIMP-1 in patients with sepsis [10,12,15,16].

In our study, no changes in MMP-9 and TIMP-1 values were observed during the first three days of ICU hospitalisation. Only on day 7 after admission to ICU, MMP-9 level was significantly higher in patients who died. This probably had a connection with the advancement of multi-organ failure. However, there was a correlation between MMP-9 and TIMP-1 on each further day of hospitalisation, i.e. higher levels of MMP-9 were accompanied by higher levels of TIMP-1, which proves that critically ill patients with multiple organ failure have effective defence mechanisms against proteolytic action of MMP-9.

The MMP-9/TIMP-1 ratio is a reflection of imbalance between MMP-9 and TIMP-1. An increase in MMP-9/TIMP-1 ratio evidences increased activity of proteolytic enzymes. In our study, this ratio on day 1 of ICU hospitalisation was 0.62 while on day 3 – 0.34. However, no differences were observed between non-survivors and survivors. A higher MMP-9/TIMP-1 ratio in the group of non-survivors was reported by Lorente et al. The absolute values were higher than 1.0 [13]. According to Chiang et al., who studied patients with extra-hospital pneumonia, the MMP-9TIMP-1 ratio was 0.28, as compared to 0.2 in healthy volunteers [17]. The MMP-9/TIMP-1 ratio seems to be useful for assessing the disease dynamics as a reflection of imbalance between the synthesis and inhibition of MMP-9. The above observations, however, require further studies in the specific group of ICU patients.

The studies concerning infections have demonstrated a tendency to increased levels of MMPs and their inhibitors. Nakamura et al., who studied 20 patients with septic shock, have observed higher levels of MMP-9 in non-survivors, as compared to survivors [18]. Yassen et al. divided the study population into two groups: patients with sepsis and patients without the features of infection and have demonstrated no differences in MMP-9 levels in both groups, although its levels were higher than those in healthy volunteers [19].

The available literature reports various MMP-9 and TIMP-1 levels. According to Hoffmann et al. [11], the level of TIMP-1 in non-survivors was 4675 ng/ml while in the study by Lorente et al. [10] this level was 797 ng/ml. In our study the level of TIMP-1 on day ‘1’ in the study population was ~340 ng/mL and the MMP-9 was ~250 ng/mL whereas in the study of Martin et al. [20] they were ~250 ng/mL and ~25 ng/mL. Those differences are likely to be caused by different laboratory kits used for determination (ELISA versus Quantibody Human Array), study population heterogeneity (mixed ICU patients versus septic subjects/ subjects with severe trauma or brain strokes) or dynamics of the inflammatory process (different CRP levels). Also, a relatively low mortality ratio in the study of Martin (i.e. 7.8% in septic and 15.4% in non-septic patients) compared to our cohort (i.e. 35.7%) needs to be taken into account in interpretation of the biomarkers.

Our results disclosed a positive correlation between TIMP-1 and the levels of lactate and creatinine and a negative correlation between TIMP-1 and haemoglobin. Moreover, a positive correlation was found between the MMP-9/ TIMP-1 ratio versus the level of lactate and platelet count. Lorente et al. have demonstrated a correlation between platelets and lactate versus MMP-9, TIMP-1 and MMP-9/ TIMP-1 ratio [10]. However, their study regarded patients with sepsis, as opposed to our group of patients admitted to the ICU with the failure of at least 2 organs, predominantly circulatory-respiratory failure.

In our study, TIPM-1 and MMP-9 / TIMP-1 ratio was a stronger predictor of 28-day mortality compared to prediction based on the APACHE scale. The APACHE scale has been the most popular ICU mortality prediction score [21].

Few limitations must be taken into account in data interpretation of our study. Firstly, the population under investigation is rather heterogeneous. Different primary diagnoses and various medical reasons of admission may influence the results. On the one hand, these short-comings should be taken into account in statistical analysis to verify possible between-group differences. On the other hand, heterogeneous clinical conditions rather than homogeneous disease states are characteristic to ICU patients. So, our results reflect the real-life scenario and further selection of critically ill subjects would only confound the conclusions. Secondly, a small size of the study population is another serious limitation and multivariable analysis was impossible to be performed. We think that further research should be carried out in a larger cohort of ICU patients to verify our findings. Nevertheless, our results seem to be adequately interesting to elucidate the issues discussed in critically ill subjects, notably because they are part of research that test the value of biomarkers as prognostic and predictive factors. Thirdly, for MMP-9, a significant increase in non-survivors has been observed after a week. No differences were found just after the ICU admission. Obviously, MMP-9 up-regulation takes time. For TIMP-1 enhanced levels were found just after admission, as well as after a week, whereas its concentrations were comparable on days ‘2’ and ‘3’. This phenomenon is difficult to explain and probably limits the prognostic value of the latter biomarker.

## 4 Conclusion

Although TIMP-1 and MMP-9 concentrations were higher in non-survivors and the MMP-9/TIMP-1 ratio was related to some parameters of critical illness, further research is needed to verify whether they can serve reliable biomarkers for early prognostication of ICU patients.
